# Supplementary material for: Role and mechanism of NCAPD3 in promoting malignant behaviors in gastric cancer
Source: Front Pharmacol. 2024 Apr 22;15:1341039. doi: 10.3389/fphar.2024.1341039 (PMC11070777; doi:10.3389/fphar.2024.1341039)
Supplement: Supplementary file 11 [file DataSheet2.ZIP › GSEA/Transcipt factor/my_analysis.Gsea.1599461814734/SUPT20H_TARGET_GENES.html]

Details for gene set SUPT20H\_TARGET\_GENES[GSEA]

|  || Dataset | filtered\_dataset.sample\_info.cls#WT\_versus\_NCAPD3\_MUT |
| Phenotype | sample\_info.cls#WT\_versus\_NCAPD3\_MUT |
| Upregulated in class | WT |
| GeneSet | SUPT20H\_TARGET\_GENES |
| Enrichment Score (ES) | 0.2748792 |
| Normalized Enrichment Score (NES) | 1.9907286 |
| Nominal p-value | 0.0 |
| FDR q-value | 0.27703658 |
| FWER p-Value | 0.66 |
Table: GSEA Results Summary

  

Fig 1: Enrichment plot: SUPT20H\_TARGET\_GENES      
 Profile of the Running ES Score & Positions of GeneSet Members on the Rank Ordered List

  

| SYMBOL | TITLE | RANK IN GENE LIST | RANK METRIC SCORE | RUNNING ES | CORE ENRICHMENT || 1 | 23248 | RPRD2 | 33 | 0.977 | -0.0096 | Yes |
| 2 | 27304 | MOCS3 | 76 | 0.844 | -0.0283 | Yes |
| 3 | 55006 | TRMT61B | 101 | 0.782 | -0.0342 | Yes |
| 4 | 50861 | STMN3 | 126 | 0.777 | -0.0401 | Yes |
| 5 | 222234 | FAM185A | 144 | 0.740 | -0.0411 | Yes |
| 6 | 51174 | TUBD1 | 153 | 0.730 | -0.0354 | Yes |
| 7 | 10795 | ZNF268 | 168 | 0.710 | -0.0347 | Yes |
| 8 | 54971 | BANP | 172 | 0.707 | -0.0255 | Yes |
| 9 | 54815 | GATAD2A | 191 | 0.685 | -0.0282 | Yes |
| 10 | 51018 | RRP15 | 192 | 0.685 | -0.0170 | Yes |
| 11 | 84190 | METTL25 | 195 | 0.684 | -0.0074 | Yes |
| 12 | 9897 | KIAA0196 | 197 | 0.682 | 0.0029 | Yes |
| 13 | 5189 | PEX1 | 200 | 0.676 | 0.0124 | Yes |
| 14 | 4836 | NMT1 | 208 | 0.669 | 0.0179 | Yes |
| 15 | 196294 | IMMP1L | 216 | 0.662 | 0.0233 | Yes |
| 16 | 57521 | RPTOR | 239 | 0.645 | 0.0168 | Yes |
| 17 | 6426 | SRSF1 | 243 | 0.637 | 0.0248 | Yes |
| 18 | 5701 | PSMC2 | 244 | 0.636 | 0.0352 | Yes |
| 19 | 9778 | KIAA0232 | 247 | 0.633 | 0.0440 | Yes |
| 20 | 528 | ATP6V1C1 | 254 | 0.625 | 0.0496 | Yes |
| 21 | 55756 | INTS9 | 257 | 0.624 | 0.0582 | Yes |
| 22 | 25974 | MMACHC | 265 | 0.621 | 0.0629 | Yes |
| 23 | 116143 | WDR92 | 267 | 0.619 | 0.0723 | Yes |
| 24 | 10944 | C11orf58 | 272 | 0.617 | 0.0792 | Yes |
| 25 | 80124 | VCPIP1 | 282 | 0.607 | 0.0822 | Yes |
| 26 | 81889 | FAHD1 | 299 | 0.592 | 0.0794 | Yes |
| 27 | 7565 | ZNF17 | 316 | 0.585 | 0.0766 | Yes |
| 28 | 27079 | RPUSD2 | 320 | 0.582 | 0.0838 | Yes |
| 29 | 94107 | TMEM203 | 333 | 0.574 | 0.0838 | Yes |
| 30 | 51491 | NOP16 | 334 | 0.573 | 0.0932 | Yes |
| 31 | 4297 | KMT2A | 337 | 0.573 | 0.1010 | Yes |
| 32 | 100529261 | CHURC1-FNTB | 343 | 0.570 | 0.1064 | Yes |
| 33 | 2965 | GTF2H1 | 356 | 0.563 | 0.1063 | Yes |
| 34 | 5810 | RAD1 | 360 | 0.560 | 0.1131 | Yes |
| 35 | 57109 | REXO4 | 370 | 0.552 | 0.1152 | Yes |
| 36 | 9786 | KIAA0586 | 379 | 0.547 | 0.1179 | Yes |
| 37 | 1387 | CREBBP | 387 | 0.544 | 0.1214 | Yes |
| 38 | 5814 | PURB | 399 | 0.536 | 0.1216 | Yes |
| 39 | 83932 | SPRTN | 404 | 0.535 | 0.1272 | Yes |
| 40 | 11019 | LIAS | 409 | 0.533 | 0.1328 | Yes |
| 41 | 147007 | TMEM199 | 410 | 0.533 | 0.1415 | Yes |
| 42 | 4771 | NF2 | 428 | 0.516 | 0.1368 | Yes |
| 43 | 148304 | C1orf74 | 447 | 0.506 | 0.1311 | Yes |
| 44 | 8607 | RUVBL1 | 451 | 0.506 | 0.1370 | Yes |
| 45 | 200933 | FBXO45 | 456 | 0.500 | 0.1421 | Yes |
| 46 | 54522 | ANKRD16 | 460 | 0.500 | 0.1479 | Yes |
| 47 | 10785 | WDR4 | 465 | 0.495 | 0.1529 | Yes |
| 48 | 146691 | TOM1L2 | 472 | 0.490 | 0.1563 | Yes |
| 49 | 84261 | FBXW9 | 491 | 0.477 | 0.1501 | Yes |
| 50 | 29101 | SSU72 | 496 | 0.475 | 0.1548 | Yes |
| 51 | 6742 | SSBP1 | 501 | 0.472 | 0.1594 | Yes |
| 52 | 54920 | DUS2 | 509 | 0.470 | 0.1616 | Yes |
| 53 | 5188 | GATB | 512 | 0.469 | 0.1677 | Yes |
| 54 | 204 | AK2 | 529 | 0.458 | 0.1628 | Yes |
| 55 | 1200 | TPP1 | 536 | 0.454 | 0.1656 | Yes |
| 56 | 29090 | TIMM21 | 537 | 0.453 | 0.1730 | Yes |
| 57 | 55527 | FEM1A | 543 | 0.449 | 0.1764 | Yes |
| 58 | 3609 | ILF3 | 544 | 0.449 | 0.1838 | Yes |
| 59 | 1819 | DRG2 | 550 | 0.447 | 0.1872 | Yes |
| 60 | 9416 | DDX23 | 553 | 0.445 | 0.1929 | Yes |
| 61 | 79080 | CCDC86 | 555 | 0.443 | 0.1994 | Yes |
| 62 | 78988 | MRPL57 | 557 | 0.441 | 0.2058 | Yes |
| 63 | 79707 | NOL9 | 560 | 0.441 | 0.2115 | Yes |
| 64 | 10773 | ZBTB6 | 562 | 0.441 | 0.2179 | Yes |
| 65 | 55245 | UQCC1 | 580 | 0.434 | 0.2118 | Yes |
| 66 | 93134 | ZNF561 | 590 | 0.431 | 0.2119 | Yes |
| 67 | 55181 | SMG8 | 602 | 0.422 | 0.2102 | Yes |
| 68 | 8021 | NUP214 | 611 | 0.417 | 0.2108 | Yes |
| 69 | 7290 | HIRA | 612 | 0.417 | 0.2176 | Yes |
| 70 | 55308 | DDX19A | 615 | 0.416 | 0.2229 | Yes |
| 71 | 10445 | MCRS1 | 617 | 0.414 | 0.2289 | Yes |
| 72 | 84948 | TIGD5 | 619 | 0.413 | 0.2348 | Yes |
| 73 | 5147 | PDE6D | 624 | 0.410 | 0.2384 | Yes |
| 74 | 55735 | DNAJC11 | 625 | 0.409 | 0.2451 | Yes |
| 75 | 10061 | ABCF2 | 626 | 0.408 | 0.2518 | Yes |
| 76 | 54663 | WDR74 | 628 | 0.408 | 0.2576 | Yes |
| 77 | 26953 | RANBP6 | 630 | 0.406 | 0.2635 | Yes |
| 78 | 80110 | ZNF614 | 642 | 0.404 | 0.2616 | Yes |
| 79 | 79807 | GSTCD | 644 | 0.402 | 0.2674 | Yes |
| 80 | 26747 | NUFIP1 | 666 | 0.394 | 0.2575 | Yes |
| 81 | 23093 | TTLL5 | 684 | 0.386 | 0.2507 | Yes |
| 82 | 55157 | DARS2 | 686 | 0.385 | 0.2562 | Yes |
| 83 | 132949 | AASDH | 689 | 0.384 | 0.2609 | Yes |
| 84 | 89910 | UBE3B | 693 | 0.381 | 0.2648 | Yes |
| 85 | 10452 | TOMM40 | 696 | 0.380 | 0.2695 | Yes |
| 86 | 10607 | TBL3 | 698 | 0.380 | 0.2749 | Yes |
| 87 | 23350 | U2SURP | 719 | 0.364 | 0.2653 | No |
| 88 | 134430 | WDR36 | 733 | 0.359 | 0.2611 | No |
| 89 | 55003 | PAK1IP1 | 734 | 0.359 | 0.2670 | No |
| 90 | 58509 | CACTIN | 748 | 0.351 | 0.2627 | No |
| 91 | 55178 | RNMTL1 | 771 | 0.337 | 0.2511 | No |
| 92 | 51154 | MRTO4 | 794 | 0.327 | 0.2394 | No |
| 93 | 79590 | MRPL24 | 800 | 0.320 | 0.2408 | No |
| 94 | 1973 | EIF4A1 | 804 | 0.319 | 0.2437 | No |
| 95 | 51373 | MRPS17 | 817 | 0.306 | 0.2394 | No |
| 96 | 9343 | EFTUD2 | 818 | 0.305 | 0.2443 | No |
| 97 | 26121 | PRPF31 | 825 | 0.301 | 0.2446 | No |
| 98 | 3146 | HMGB1 | 831 | 0.295 | 0.2456 | No |
| 99 | 2224 | FDPS | 841 | 0.273 | 0.2430 | No |
| 100 | 3309 | HSPA5 | 876 | -0.288 | 0.2214 | No |
| 101 | 54968 | TMEM70 | 879 | -0.291 | 0.2246 | No |
| 102 | 23731 | TMEM245 | 891 | -0.306 | 0.2211 | No |
| 103 | 9181 | ARHGEF2 | 902 | -0.326 | 0.2187 | No |
| 104 | 440026 | TMEM41B | 932 | -0.355 | 0.2021 | No |
| 105 | 53349 | ZFYVE1 | 1018 | -0.417 | 0.1431 | No |
| 106 | 54676 | GTPBP2 | 1050 | -0.447 | 0.1264 | No |
| 107 | 253959 | RALGAPA1 | 1066 | -0.456 | 0.1222 | No |
| 108 | 196394 | AMN1 | 1079 | -0.461 | 0.1204 | No |
| 109 | 100534599 | ISY1-RAB43 | 1082 | -0.462 | 0.1264 | No |
| 110 | 10001 | MED6 | 1190 | -0.561 | 0.0528 | No |
| 111 | 57649 | PHF12 | 1212 | -0.588 | 0.0461 | No |
| 112 | 22931 | RAB18 | 1231 | -0.608 | 0.0421 | No |
| 113 | 145508 | CEP128 | 1236 | -0.614 | 0.0490 | No |
| 114 | 5187 | PER1 | 1254 | -0.641 | 0.0463 | No |
| 115 | 11057 | ABHD2 | 1258 | -0.646 | 0.0545 | No |
| 116 | 7994 | KAT6A | 1279 | -0.666 | 0.0499 | No |
| 117 | 57157 | PHTF2 | 1334 | -0.755 | 0.0205 | No |
| 118 | 26959 | HBP1 | 1395 | -1.073 | -0.0085 | No |
| 119 | 3329 | HSPD1 | 1402 | -1.183 | 0.0062 | No |
Table: GSEA details [plain text format]

  

Fig 2: SUPT20H\_TARGET\_GENES      
 Blue-Pink O' Gram in the Space of the Analyzed GeneSet

  

Fig 3: SUPT20H\_TARGET\_GENES: Random ES distribution      
 Gene set null distribution of ES for **SUPT20H\_TARGET\_GENES**
